# Optogenetic Tools for Spatiotemporal Interrogation of Cytoskeletal Dynamics

**DOI:** 10.1021/acs.bioconjchem.6c00071

**Published:** 2026-03-26

**Authors:** Danica T. Du, Anna Price, Tien-Hung Lan, Yubin Zhou

**Affiliations:** † Center for Translational Cancer Research, Institute of Biosciences and Technology, College of Medicine, 14736 Texas A&M University, Houston, Texas 77030, United States; ‡ Department of Translational Medical Sciences, College of Medicine, Texas A&M University, Houston, Texas 77030, United States

## Abstract

The cytoskeleton is a dynamic intracellular network that governs cell shape, migration, division, and mechanotransduction. Precise spatiotemporal control of cytoskeletal regulation is essential for understanding how these processes are coordinated in physiology and disease, yet conventional pharmacological and genetic approaches often lack sufficient resolution or reversibility. Optogenetic technologies provide a powerful alternative by enabling light-controlled, noninvasive manipulation of cytoskeletal regulators with high temporal precision and subcellular specificity. This review summarizes recent advances in genetically encoded optogenetic tools for interrogating cytoskeletal dynamics. We discuss core design strategies, including allosteric regulation, light-induced oligomerization, heterodimerization, and dissociation, and highlight representative applications targeting actin filaments, microtubules, and upstream signaling pathways such as Rho family GTPases. We conclude by outlining current limitations and emerging directions, including improved tissue penetration, reduced phototoxicity, and multiplexed optical control, which are expected to further expand the utility of optogenetics in cytoskeleton research.

## Graphical abstract

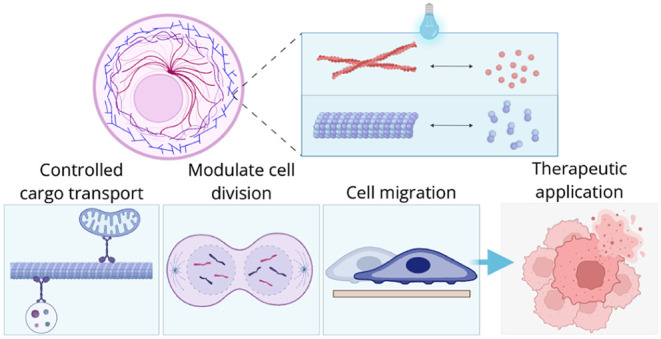

## 1 Introduction

The cytoskeleton is composed of three filament systems: actin filaments, microtubules, and intermediate filaments.^1,2^ Together, they form a highly organized yet dynamic network that coordinates a variety of cellular functions. Through tightly regulated cycles of polymerization and depolymerization, the cytoskeleton reorganization governs cell structures, mediates the trafficking of macromolecules and organelles, promotes cell migration and division, and maintains mechanical resilience. Beyond its structural role, cytoskeleton reorganization is also an integral component of signal transduction and underpins diverse biological processes, including embryonic development, tissue morphogenesis, immune cell activation, and muscle contraction.^1^ Dysregulation of cytoskeleton dynamics is directly implicated in pathological conditions such as cancer invasion and metastasis, as well as neurodegenerative diseases.^3,4^ Accordingly, approaches that enable precise spatiotemporal manipulation of the cytoskeleton provide powerful means to interrogate its regulatory mechanisms, facilitating the study ofboth normal cellular physiology and disease processes.

Historically, studies on cytoskeleton dynamics have relied heavily on pharmacological agents and genetic perturbations.^5^ While these approaches have yielded invaluable insights, they are intrinsically constrained by limited temporal precision, irreversibility, and a lack of subcellular specificity. For example, widely used actin-disrupting drugs, such as latrunculin B and cytochalasin D, effectively perturb filament assembly by sequestering actin monomers or capping filament ends, respectively. However, their global and sustained effects on the cytoskeleton often mask localized or transient regulatory events. Similarly, commonly used microtubule-targeting agents, including paclitaxel and vincristine, induce mitotic arrest and apoptosis through stabilizing or destabilizing microtubules, but do not permit reversible or spatially confined modulation.^6–8^ In parallel, genetic knockouts or knockdowns frequently lead to cell death or induce compensatory adaptations, limiting their ability to capture rapid and context-dependent cytoskeletal responses.

In recent years, optogenetic tools have emerged as powerful tools for cytoskeletal research, enabling light-controlled and noninvasive manipulation of cytoskeleton organization with exceptional temporal and spatial precision.^4,9^ By coupling cytoskeleton regulators to light-responsive domains derived from plants, bacteria, or fungi, optogenetic systems allow repeatable perturbations of the cytoskeleton on timescales ranging from milliseconds to minutes at subcellular resolution.^4,10,11^ These capabilities allow direct causal interrogation of cytoskeleton dynamics in living cells and organisms, providing a platform to link molecular regulatory mechanisms with cellular behaviors. Optogenetic approaches have thus substantially expanded the experimental toolkit for studying cytoskeleton organization, mechanics, and function under physiologically relevant conditions. This review highlights recent advances in genetically encoded optogenetic systems for cytoskeleton manipulation and discusses their underlying design principles.

## 2 Design Principles of Optogenetic Manipulation of the Cytoskeleton

Similar to other optogenetic tools, cytoskeleton modulating systems consist of a photosensory module (PSM), typically derived from the light-responsive domain of a plant or bacterial photoreceptor, and an effector domain that can be expressed either as a fusion with the PSM or as an independent protein.^4,11,12^ A diverse set of photosensory modules has been extensively characterized for regulating protein function in response to light.^4,10–18^ Despite variations in PSM and effector domain combinations, optogenetic tools for cytoskeleton manipulation generally operate through four principal mechanisms:^4,12^ allosteric switching, homo-oligomerization, heterodimerization, and dissociation (Figure 1). By coupling effector domains with PSMs bearing distinct kinetics, reversibility, activation/deactivation wavelengths, and photosensitivities, these design strategies enable versatile manipulation across a broad range of biological applications (Table 1).

**Figure 1:**
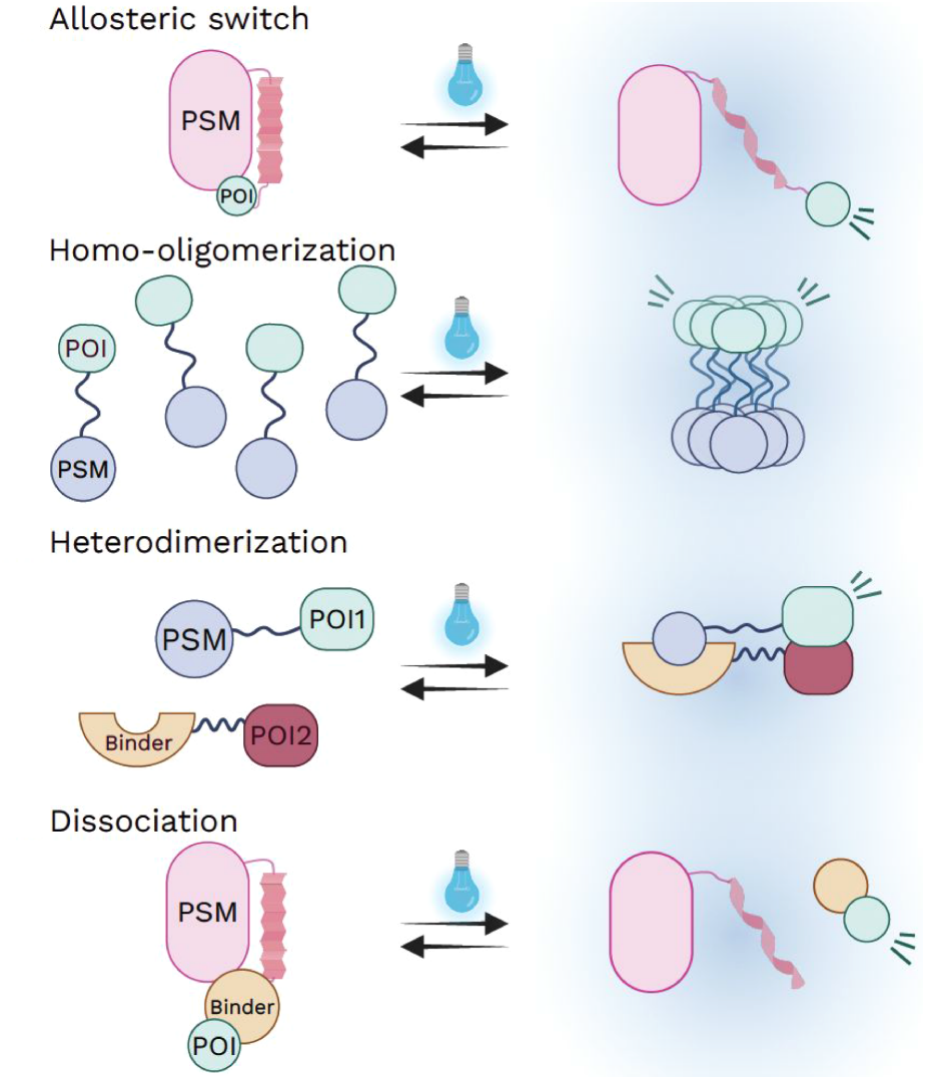
Schematic overview of the four mechanistic principles of optogenetics. (A) Allosteric switch: Light-induced conformational changes in the PSM expose previously caged functional motifs, thereby modulating effector activity. (B) Homo-oligomerization: Light stimulation drives dimerization or higher-order clustering of PSM-fused effectors, increasing local concentration and binding avidity to activate downstream signaling pathways. (C) Heterodimerization: Light illumination promotes interaction between complementary binding partners, enabling recruitment of effector domains to user-defined subcellular regions or reconstitution of split protein fragments into functional assemblies. (D) Dissociation: Light exposure destabilizes protein–protein interactions, resulting in dissociation of complexes and release or activation of the fused effector domain.

**Table 1:** Commonly Used Photosensitive Domains in Cytoskeletal Manipulation

Excitation wavelength	Photosensory module (PSM)	PSD variants	Mechanism of action
Blue light (450 nm)	Light-oxygen-voltage (LOV)	*Rs*LOV (from *Rhodobacter sphaeroides*)	Dissociation of dimer to monomer upon exposure to light.^20^
		*As*LOV2 (from *Avena sativa*)	Light-induced conformational changes of *As*LOV2 allosterically exposes the protein of interest (POI) fused to it.^21^
		LOVTRAP (derived from LOV2)	LOV2 selectively binds to an engineered small protein Zdk that sequesters the POI from its effectors in the dark. Light induces the dissociation of Zdk from LOV2, freeing the POI to move to its site of action.^22^
		iLID-sspB (derived from *As*LOV2	Light-induced heterodimerization of SspB protein and SsrA peptide caged by AsLOV2.^31^
		Vivid or VVD (from *Neurospora crassa*)	Formation of a homodimer from monomer by light.^28^
		Magnet (derived from VVD)	Light-induced fast heterodimerization of the pMag-nMag protein pair.^32^
	Cryptochorme2 (CRY2)	CRY2/CIB1 or CYR2-PHR/CIBN	Light-induced homo- or heterodimerization of CRY2 and CIB1 or their truncated versions, CYR2-PHR and CIBN.^30,33,54^
Cyan light (500 nm) for excitation and violet light (400 nm) for reversion	Photodissociable dimeric Dronpa (pdDronpa)	Derived from green fluorescent protein (GFP)	A dimer in the dark is converted by cyan light a monomer that can be reversed back to a dimer by violet light.^34^
Red light (660 nm) for excitation and Near-infrared light (NIR) (740 or 780 nm) or dark for reversion	Phytochrome	PhyB/PIF3 or PIF6 (from *Arabidopsis thaliana*)	Light-regulated heterodimerization of PhyB with its binding partner PIF3 or PIF6.^39^
	Bacterial phytochromes (BphPs)	*Rp*BphP1/PpsR2 or QPAS1 (from *Rhodopseudomonas palustris*)	Light-regulated heterodimerization of PhyB with its binding partner PpsR2 or QPAS1 (a smaller engineered variant of PpsR2).^37,38^

Allosteric switching designs exploit light induced conformational changes within a photosensory module to regulate effector activity. Among the most widely used PSMs are light-oxygen-voltage (LOV) domains derived from microbial and plant proteins, such as *Rs*LOV from*Rhodobacter sphaeroides*and *As*LOV2 from*Avena sativa*.^19^ These domains sense blue light through a conserved flavin based photochemical reaction.^20–22^ Upon illumination, a flavin cofactor, either flavin mononucleotide (FMN) or flavin adenine dinucleotide (FAD), forms a transient covalent adduct with a conserved cysteine residue within the LOV domain, initiating conformational changes in the protein scaffold (Figure 1A). In allosteric designs, this light induced structural change is harnessed to modulate effector protein interactions or to expose previously caged functional motifs, thereby enabling reversible and precise control of protein activity.^4,23–27^

Homo-oligomerization based designs rely on light-induced self-association of PSMs to regulate effector function. Upon illumination, PSMs undergo dimerization or higher-order clustering, which can increase local concentration and binding avidity of effector domains, thereby activating downstream signaling pathways (Figure 1B). Representative examples include the Vivid (VVD) protein, which undergoes light-dependent homodimerization using FAD as a chromophore,^28^ and cryptochrome 2 (CRY2), a FAD binding photoreceptor that forms light-induced oligomers. Engineered CRY2 variants further enhance this strategy by providing faster or slower ON/OFF kinetics and improved control over clustering behavior.^29,30^

Heterodimerization based designs employ light-induced association between two distinct protein components to control effector localization or activity (Figure 1C). In these systems, illumination promotes interaction of complementary binding partners, enabling recruitment of effector domains to user-defined subcellular regions or reconstitution of split protein fragments into functional assemblies. In the light-induced dimer (iLID)-SspB system, the SsrA peptide is sterically caged within the AsLOV2 scaffold in the dark, preventing its interaction with SspB. Upon blue light illumination, a conformational change in AsLOV2 exposes the SsrA peptide, permitting high affinity binding to SspB and resulting in rapid recruitment of SspB fused effector domains to the illuminated region.^31^ The Magnet system, which was evolved from the VVD, uses complementary pMag and nMag proteins to achieve similar light-dependent recruitment with faster dark state dissociation kinetics.^32^ In addition, CRY2 also supports heterodimerization with its binding partner CIB1 or with the truncated CIBN.^33^

Light-controlled dissociation designs regulate effector activity through light-induced separation of preassociated protein complexes (Figure 1D). In this strategy, light exposure destabilizes protein–protein interactions, resulting in release or activation of the fused effector domain. The photodissociable dimeric Dronpa (pdDronpa), derived from green fluorescent protein, undergoes reversible dissociation in response to cyan and violet light.^34^ The LOV2 and Zdk^22,35^ system similarly employs light-induced conformational changes in the LOV2 domain to disrupt its interaction with the Zdk binding partner, thereby releasing Zdk fused effector proteins in a reversible manner. The recently developed PhoBIT^36^ system further extends this dissociation-based strategy by operating on an engineered, compact seven amino acid ssrA tag. In PhoBIT1, a light-induced conformational change in LOV2 allosterically modulates the ssrA binding pocket of sspB, triggering dissociation of the ssrA tag and functioning as a light controlled OFF switch.^36^

To achieve efficient light penetration in thick tissues and *in vivo* settings, near-infrared (NIR) light responsive PSMs have also been developed, most prominently phytochromes from*Arabidopsis thaliana*phytochrome and bacterial phytochromes (BphPs).^37–39^ These systems use phycocyanobilin or biliverdin IXα as chromophores and reversibly isomerize between red and far-red light-absorbing states to regulate enzymatic activity or biomolecular interactions with reduced scattering and phototoxicity compared to blue light systems. The availability of near-infrared optogenetic modules expands the applicability of cytoskeleton manipulation beyond cultured cells, enabling spatially resolved control in complex tissues and whole organisms.

Together, these four design principles, allosteric switching, homo oligomerization, heterodimerization, and light controlled dissociation, define a versatile and modular framework for optogenetic manipulation of the cytoskeleton. By selecting appropriate photosensory modules and interaction architectures (Table 1), researchers can tailor the kinetics, reversibility, spatial precision, and wavelength dependence of cytoskeletal perturbations to match specific biological questions. This design space has enabled increasingly sophisticated control over cytoskeletal organization and dynamics, providing powerful tools to dissect the causal relationships between cytoskeletal regulation and cellular function in both physiological and pathological contexts.

## 3 Optogenetic Modulation of Rho GTPase Signaling

The Rho family of small GTPases comprises central regulators of cytoskeletal organization, functioning as molecular switches that coordinate actin and microtubule dynamics in space and time.^40,41^ By cycling between active GTP-bound and inactive GDP-bound states, Rho GTPases such as Rac1, Cdc42, and RhoA regulate actin polymerization and contractility through effectors such as formins, Arp2/3 complexes, and Rho associated kinases, while also modulating microtubule stability and cortical interactions via microtubule associated and plus end tracking proteins.^42–45^ Through these coordinated actions, Rho GTPases integrate extracellular cues to drive cytoskeleton remodeling underlying cell polarity, migration, division, and morphogenesis. Dysregulation of Rho GTPase signaling disrupts both actin and microtubule networks and is implicated in developmental disorders, neurological disease, and cancer progression.^3,7,8^ Their rapid kinetics, switch like behavior, and strong signal amplification make Rho GTPases particularly well suited for optogenetic manipulation, enabling precise spatiotemporal control of cytoskeleton reorganization in living cells.

One of the earliest examples of optogenetic modulation of Rho GTPases was exemplified by the engineering of photoactivatable Rac1 (PA-Rac1).^23,46^ In this design, the LOV2 domain was fused to the N-terminus of a constitutively active Rac1 (Q61L), enabling steric inhibition of effector binding in the dark and rapid activation upon blue light illumination. Light-induced conformational changes in LOV2 relieved this inhibition, allowing Rac1 to engage downstream effectors and drive actin reorganization, membrane protrusion, and changes in cell morphology and motility (Table 2, Figure 2A).^23,46^ This work provided early proof that optogenetic control of a single Rho GTPase is sufficient to elicit robust cytoskeletal responses with high spatial and temporal precision.

**Table 2:** Optogenetic Tools for Modulating Cytoskeleton Dynamics

Optogenetic Tool	Module	Mechanism	Wavelength (nm)	Design Principle	Cytoskeleton Application	Reference
PA-Rac1	LOV2	Allosteric	On: 450; Off: dark	LOV2 Jα helix sequesters Rac1; light leads a conformational change uncaging Rac1.	Actin reorganization, membrane protrusion, and changes in cell morphology and motility	^9,23,70^
OptoGEF-RhoA	CRY2/CIBN	Heterodimerization	On: 450; Off: dark	Light triggers the binding of CRY2/CIBN, recruiting ARHGEF11.	Contraction, tension, tissue compaction	^52^
Z-lock (cofilin)	LOV2/Zdk	Dissociation	On: 450; Off: dark	Light causes the release of Zdk from LOV2, activating cofilin.	Actin disassembly/protrusions	^57^
Z-lock (αTAT)	LOV2/Zdk	Dissociation	On: 450; Off: dark	Light causes the release of Zdk from LOV2, activating αTAT.	Microtubules acetylation	^57^
CRY2olig-Nck	CRY2	Oligomerization	On: 450 Off: dark	CRY2olig fused to Nck SH3 domain, clustering upon light exposure.	Actin polymerization	^42^
OptoVCA	iLID/SspB	Heterodimerization	On: 450; Off: dark	Light induces binding between iLiD and SspB, trigger recruitment of WAVE1-VCA to plasma membrane.	Actin nucleation	^56^
Opto-katanin	iLID/SspB VVD	Oligomerization/Heterodimerization	On: 450; Off: dark	Light induces dimerization of VVD and iLID/SspB, leading to assembly of katanin and its targeting to microtubule.	Microtubule disassembly, cytoskeleton remodeling	^55^
Opto-spastin	CRY2/CIBN	Oligomerization	On: 450 Off: dark	Light induces oligomerization of CRY2-fused spastin and its binding to CIBN.	Microtubule severing	^59^
OptoTIP-SAW	CRY2	Oligomerization	On: 450; Off: dark	CRY2-SxIP-SAW construct clusters and targets to microtubule with light stimulation.	Microtubule severing	^49^
π-EB1	LOV2/Zdk	Dissociation	On: 450; Off: dark	LOV2 and Zdk are bound, forming a functional EB1 with a zipper in the dark. Light-induced dissociation disrupts the complex.	Microtubule destabilization	^60^

**Figure 2:**
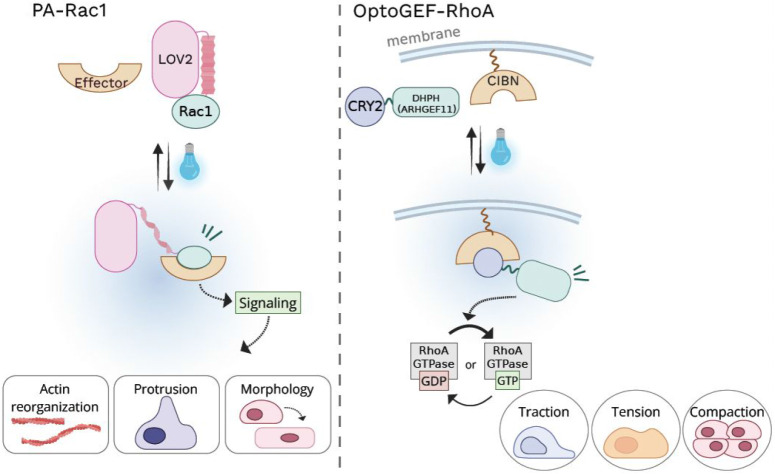
Optogenetic modulation of Rho GTPase signaling. Optogenetic modulation of Rho GTPase signaling. Left, PA-Rac: light-induced conformational change in LOV2 exposes the constitutively active Rac1 Q61L, allowing it to engage downstream effectors and drive actin polymerization. Right, optoGEF-RhoA: light stimulation recruits CRY2-fused ARHGEF11 (optoGEF-RhoA) to the plasma membrane-anchored CIBN, enabling localized activation of RhoA signaling.

While direct allosteric or steric gating of small GTPases offers rapid and robust control, its generalization across the Rho family often requires case-specific optimization of the LOV2-small GTPase interface. Consequently, subsequent efforts shifted toward targeting the upstream regulatory machinery that governs endogenous Rho GTPase activity. Rho GTPases cycle between an active GTP-bound state and an inactive GDP-bound state, a process regulated by guanine nucleotide exchange factors (GEFs) and GTPase-activating proteins (GAPs).^47,48^ Optogenetic recruitment or release of these regulators provides a modular and broadly applicable strategy for controlling Rho GTPase signaling without directly modifying the GTPase itself.

A series of optogenetic tools have therefore been developed to locally activate Rho GTPases through light-controlled membrane recruitment of GEFs.^12,17,49^ Using heterodimerization systems such as iLID and SspB, exchange factors for Rac1, Cdc42, or RhoA are fused to iLID, while SspB is tethered to membrane targeting motifs.^31,50^ Light-induced recruitment of these exchange factors enables spatiotemporally precise activation of endogenous GTPases and has been widely used to study actin polymerization, cell polarity, and directed migration.^51–53^ Similarly, the CRY2 and CIBN system has been used to recruit CRY2-fused exchange factors such as ARHGEF11 (optoGEF-RhoA) to the plasma membrane- or mitochondria-anchored CIBN, enabling localized activation or deactivation of RhoA signaling that couples to cellular traction, intercellular tension and tissue compaction (Figure 2B).^52^ In addition, the LOV2 and Zdk based system has also been used to sequester Rac specific exchange factors such as Vav2 at intracellular membranes and release them upon illumination, providing reversible control over Rac signaling amplitude and localization.^22,27,54^ Together, these approaches illustrate how optogenetic regulation of Rho GTPase signaling can be tuned to achieve graded and reversible control of cytoskeleton dynamics in a light-dependent manner.

Collectively, light-controlled manipulation of Rho GTPase signaling has established a versatile framework for probing cytoskeletal regulation. These approaches illustrate how modulating Rho GTPase signaling can be tuned to drive actin polymerization and contractibility to establish cell polarity, directional migration, and regulate mechanical tension through optical control. Importantly, optogenetic perturbations have enabled researchers to dissect causal relationships between localized signaling events and emergent cellular behaviors, including how spatial gradients of Rac1 activity control membrane protrusion and how RhoA-driven contractility regulates tissue morphogenesis and epithelial tension. By operating at the level of upstream signaling nodes, these tools leverage endogenous amplification mechanisms and preserve native downstream wiring, enabling robust cytoskeletal responses with minimal perturbation. At the same time, the diversity of optogenetic designs summarized in recent toolkits underscores important trade-offs between speed, reversibility, spatial confinement, and pathway specificity, which must be considered when selecting strategies to interrogate actin and microtubule dynamics.

## 4 Direct Optogenetic Manipulation of the Polymerization of the Cytoskeleton

Direct optogenetic manipulation of cytoskeletal dynamics provides a complementary strategy to upstream signaling control by acting directly on filament assembly and disassembly. Unlike pathway-level perturbations, these approaches minimize indirect effects and enable precise interrogation of cytoskeletal mechanics through light-controlled caging, recruitment, or oligomerization of filament associated regulators.

### 4.1 Actin

Optogenetic control of actin disassembly has been achieved by directly manipulating actin-severing and destabilizing factors.^55,56^ A prominent strategy employs the Z-lock system, in which LOV2 and Zdk form an intramolecular bridge that sterically cages an effector in the dark and releases it upon illumination. This approach was first applied to the actin severing protein cofilin (Z-lock cofilin), where light-induced uncaging triggered rapid filamentous actin (F-actin) disassembly and cytoskeletal remodeling (Figure 3A).^57^ In addition to the allosteric/steric gating strategy, light-controlled nucleocytoplasmic shuttling has also been harnessed to engineer the optogenetic cofilin-1 (opto-cofilin). In this system, light-induced nuclear export of opto-cofilin stabilizes nuclear F-actin during mitotic exit, whereas termination of illumination triggers rapid reorganization and subsequent disassembly of nuclear F-actin.^58^

**Figure 3:**
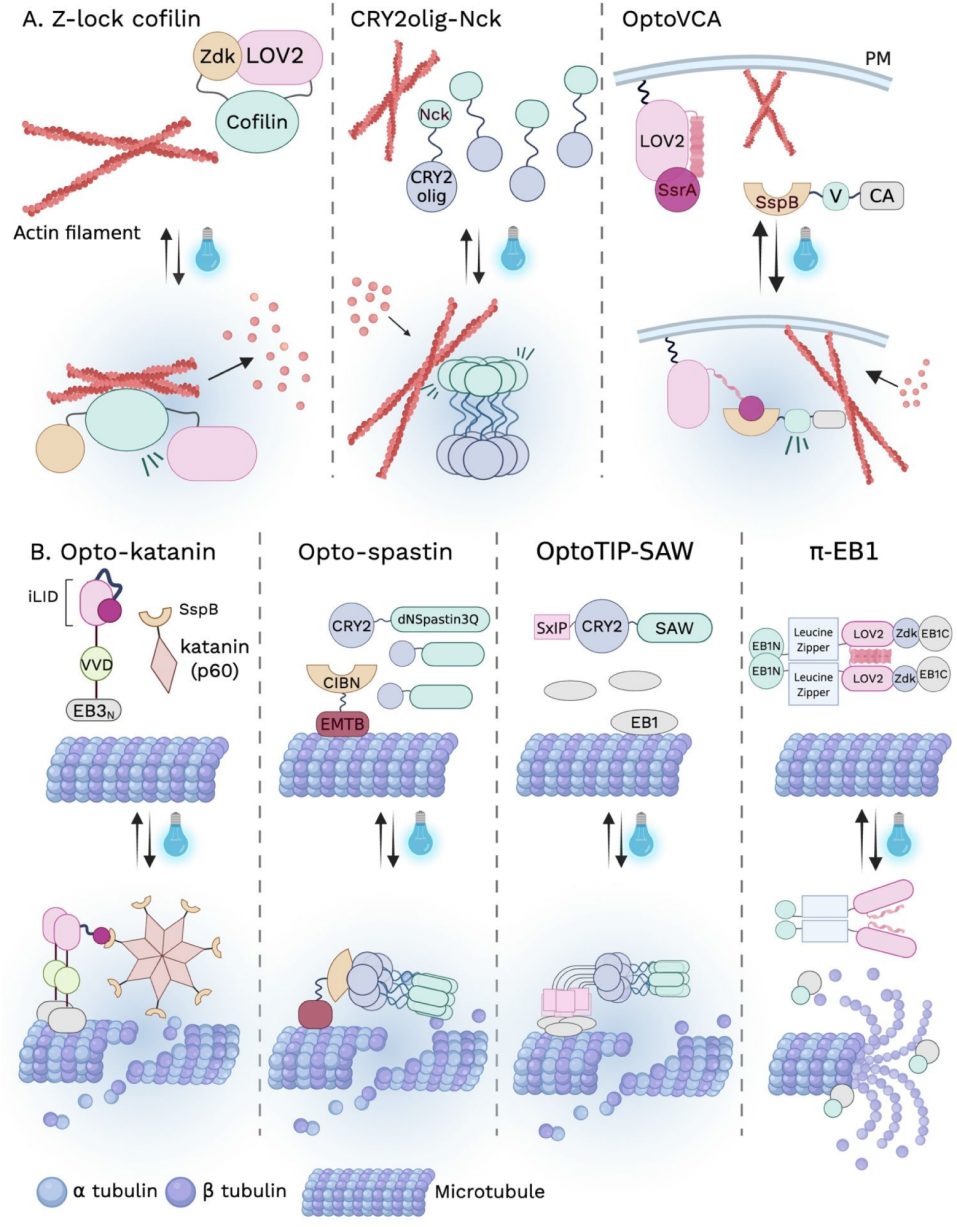
Direct optogenetic modulation of cytoskeleton dynamics. (A) Optogenetic modulation of actin dynamics. Left, Z-lock cofilin: LOV2 and Zdk form an intramolecular bridge that sterically cages cofilin; upon illumination, light-induced dissociation releases cofilin, triggering rapid F-actin disassembly. Middle, CRY2olig-Nck: the three SH3 domains of Nck fused to CRY2olig exhibit diffuse localization in the dark but undergo rapid clustering upon blue light exposure, leading to the formation of actin clusters that colocalize with Nck assemblies. Right, OptoVCA: To control the intracellular localization and local density of the VCA domain, iLID was fused to the plasma membrane anchor stargazin and SspB was fused to the VCA domain of WAVE1. Light-induced recruitment of the VCA domain to the plasma membrane promotes Arp2/3-dependent actin polymerization and formation of the cortical actin network. (B) Optogenetic modulation of microtubule dynamics. Opto-katanin: opto-katanin consists of two components: a microtubule anchor containing the EB3 microtubule-binding domain (EB3N) fused to VVD and iLID, and the p60 subunit of katanin fused to SspB. Upon blue light illumination, VVD homodimerization enhances EB3N binding to microtubules, while concurrent iLID unfolding permits recruitment of SspB-p60, resulting in localized katanin activation and microtubule disassembly. Opto-spastin: opto-spastin also comprises two components: a microtubule-binding-deficient spastin mutant (dNSpastin3Q) fused to CRY2, and the microtubule-binding domain of ensconsin (EMTB) fused to CIBN. Local blue light illumination drives CRY2-CIBN heterodimerization, recruiting dNSpastin3Q to microtubules and inducing microtubule disassembly specifically within illuminated regions. OptoTIP-SAW: a catalytically competent but microtubule-binding-deficient spastin fragment (residues 228–616, K3/Q3, designated SAW) is fused to a CRY2-coupled SxIP motif. Light-induced CRY2 oligomerization mimics the native multivalent SxIP assembly within plus-end tracking protein (+TIP) complexes, enhancing EB1 recruitment and microtubule tip-tracking while simultaneously reconstituting spastin activity, together leading to efficient severing of microtubule plus-end structures. π-EB1: π-EB1 comprises two components: the N-terminal CH domain of EB1 fused to a GCN4 leucine zipper and LOV2, and the C-terminal EBH domain of EB1 fused to Zdk1. Blue light illumination disrupts the LOV2-Zdk1 interaction and dissociates π-EB1, thereby leading to rapid + TIP complex disassembly and acute attenuation of microtubule growth.

In contrast to strategies that promote F-actin disassembly, several optogenetic tools have been developed to actively drive actin polymerization by increasing the local concentration of nucleation-promoting factors.^30,42,56^ Actin polymerization is initiated by various nucleation-promoting factors such as Neural Wiskott–Aldrich Syndrome Protein (N-WASP) and WASP-family verprolin homologous protein 1 (WAVE1), which in turn activate the Arp2/3 complex to nucleate branched actin networks. In early implementations, CRY2 was fused to the Verprolin, Central, Acidic (VCA) domain of N-WASP or to SH3 domain of the adaptor protein Nck. In the dark, these constructs were diffusely distributed, whereas blue light-induced CRY2 oligomerization clustered the VCA or Nck domain, leading to actin polymerization (Figure 3A).^30^ This work demonstrates that light-controlled clustering of nucleation-promoting factors is sufficient to promote actin polymerization. More recently, the OptoVCA system employed the iLID and SspB pair to reversibly control the localization of the WAVE1 VCA domain, which enables spatially confined actin polymerization at defined membrane regions (Figure 3A).^56^

### 4.2 Microtubule

Optogenetic manipulation of microtubule depolymerization has been achieved by controlling microtubule severing enzymes or essential plus-end regulators.^55,59^ Recruitment-based strategies have used heterodimerization systems such as CRY2 and CIBN or iLID and SspB to translocate engineered severing proteins, including spastin and katanin, from the cytosol to microtubules labeled by specific microtubule-associated proteins, resulting in rapid microtubule fragmentation and collapse of the filament network upon illumination (Figure 3B).^55,59^ More recently, single component strategies have been developed to induce microtubule disassembly through light-controlled oligomerization. In the OptoMT-SAW and OptoTIP-SAW systems, a truncated spastin fragment (residues 228-616) is fused to CRY2-coupled microtubule lattice-binding or plus end tracking modules (+TIP), enabling light-induced CRY2 oligomerization to reconstitute functional hexameric spastin and promote microtubule destabilization (Figure 3B).^49^ In addition to targeting microtubule severing enzymes, optogenetic control has also been achieved by manipulating end-binding protein 1 (EB1), a core component of + TIP complexes required for microtubule growth.^60^ EB1 functions as a dimer, with an N-terminal calponin homology domain that binds to growing microtubule plus ends and a C-terminal domain that recruits additional +TIP proteins (Figure 3B).^60^ Using the LOV2 and Zdk system, EB1 was split into two light-controllable fragments that reassemble into a functional protein in the dark. Blue light illumination induces dissociation of EB1 from growing microtubule plus ends, thereby disrupting plus end complexes and promoting microtubule disassembly.^60^ By directly controlling cytoskeleton assembly and disassembly, these optogenetic tools have allowed researchers to dissect how microtubular dynamics contribute to cytoskeletal remodeling and cellular architecture. For example, light-controlled perturbations of microtubule severing enzymes have revealed how localized microtubule turnover regulates cell polarity and directional migration, while optogenetic disruption of + TIP complexes has provided insights into how microtubule growth dynamics influence intracellular trafficking and cytoskeletal coordination.

## 5 Optogenetic Manipulation of Cytoskeleton Modifications and Cargo Transport

Optogenetic manipulation has been applied to regulate post-translational modifications of tubulin, which serve as key molecular codes for microtubule mechanics and motor interactions.^9,61^ Among these, tubulin acetylation and detyrosination are closely associated with increased microtubule stability, altered lattice flexibility, and selective recruitment of motor proteins and microtubule-associated proteins. The LOV2 and Zdk system was first used to cage α-tubulin acetyltransferase (αTAT), an enzyme that catalyzes the acetylation of α-tubulin at the position K40 and enhances microtubule stability.^57^ Light-induced uncaging of αTAT resulted in rapid and spatially confined increases in microtubule acetylation. Beyond acetylation, optogenetic approaches have also been extended to other tubulin modifications, including detyrosination. A recent study demonstrated that light-controlled activation of vasohibin 1 (VASH1), an enzyme that regulates the tubulin tyrosination cycle, enables optogenetic control of microtubule detyrosination, providing a powerful strategy to dissect how tubulin modification states shape cytoskeletal organization and intracellular transport.^49,62^

In addition to directly modulating cytoskeletal structure and post-translational modifications, optogenetic tools have been widely applied to manipulate cytoskeleton-dependent cargo transport and force generation mediated by motor proteins.^63,64^ Motor proteins such as kinesin, dynein, and myosin transport vesicles and organelles along actin filaments or microtubules and generate mechanical forces that contribute to cell polarity, intracellular organization, and tissue architecture.^63,65^ A wide range of optogenetic strategies have been developed to control cargo transport by light-controlled recruitment of motor domains to specific cargos or subcellular compartments.^63,64^ These approaches typically rely on homo-oligomerization and heterodimerization systems, including iLID and SspB, CRY2 and CIBN, or LOV based interaction pairs, to reversibly couple motor proteins to vesicles, organelles, or protein scaffolds in a spatiotemporally defined manner.

Using these systems, optogenetic recruitment of kinesin or dynein motors has enabled directional transport of cargos such as peroxisomes, endosomes, mitochondria, and lysosomes, allowing precise control over cargo positioning and redistribution within cells.^64,66,67^ Beyond vesicular transport, optogenetic coupling of motors to cytoskeletal elements themselves has been used to probe force-mediated reorganization.^68,69^ For example, light-controlled recruitment of kinesin to intermediate filaments has been shown to perturb vimentin network organization, revealing mechanical coupling between microtubules and intermediate filaments.^68,69^ These previous studies illustrate how motor-driven transport influences cytoskeletal organization and coordination of intracellular architecture. Collectively, these cargo transport tools have provided powerful means to dissect how motor-driven transport and force generation contribute to cellular organization and dynamics.

## 6 Conclusion and Future Perspectives

The convergence of optical technologies and protein engineering has opened new avenues for precise interrogation of the cytoskeleton. Optogenetic tools have been successfully applied in the studies of cytoskeleton dynamics and their associated cellular and physiological processes.^9^ To date, the majority of optogenetic tools used to manipulate cytoskeletal processes rely on blue-light photoreceptors, including light-oxygen-voltage (LOV) domains, cryptochromes, and VVD-derived modules. One important reason for this prevalence is that these photoreceptors utilize flavin-based chromophores (FMN or FAD) that are naturally present in mammalian cells. As a result, these systems can function as fully genetically encoded tools without requiring exogenous cofactors or additional biosynthetic pathways. Moreover, blue-light responsive photoreceptors have been extensively characterized and engineered over the past decade, providing diverse modules with tunable activation kinetics, reversibility, and modular interaction architectures. These properties have facilitated the rapid development of optogenetic platforms for controlling cytoskeletal regulators, making blue-light systems the dominant framework in current cytoskeleton optogenetics studies.

Nevertheless, important limitations persist that require further optimization. A key remaining issue is phototoxicity, particularly in long-term imaging or high-power illumination. The development of tools responsive to far-red or near-infrared light, such as BphPs, offers a promising direction.^37–39^ Another advantage of these red-shifted optogenetic tools is the deeper light penetration of animal tissues in *in vivo* studies. However, the broader implementation of far-red optogenetic systems remains challenging because many require exogenous chromophores such as phycocyanobilin, which are not naturally produced in mammalian cells and must therefore be supplemented or introduced through additional metabolic engineering strategies. To enhance tissue penetration, combining optogenetic tools with other engineering approaches such as multiphoton-responsive tools, upconversion nanoparticles, or wireless optoelectronics, may also further improve the utility of current existing optogenetic systems *in vivo* applications.^4,11,14^ The currently available optogenetic systems also lack the multiplexing required for dissecting complex signaling networks. Spectral overlap and undesired crosstalk remain a challenge in current optogenetic tools. Development of truly orthogonal optogenetic pairs and fast-switching photochromic actuators will expand control in complex systems.

The development of innovative optogenetic tools has provided opportunities to explore many complex questions that cannot be addressed with conventional approaches. Future efforts should focus on integrating new engineering devices, machine-learning-guided feedback loops, and organoid models to dissect cytoskeletal regulation in three-dimensional environments. In addition to basic research, applying optogenetic tools to precisely target the cytoskeleton in various diseases, including cancer and neurodegenerative disorders, offers new therapeutic potential.^3,4^ The ability to spatiotemporally control cytoskeletal dynamics or specific cytoskeletal components provides opportunities for targeted perturbation of diseased cells while minimizing unintended effects on neighboring normal cells, including in tumor and Alzheimer’s disease contexts.
